# Supplementary figures and images for: Iron trafficking in patients with Indian Post kala-azar dermal leishmaniasis
Source: PLoS Negl Trop Dis. 2020 Feb 5;14(2):e0007991. doi: 10.1371/journal.pntd.0007991 (PMC7001907; doi:10.1371/journal.pntd.0007991)

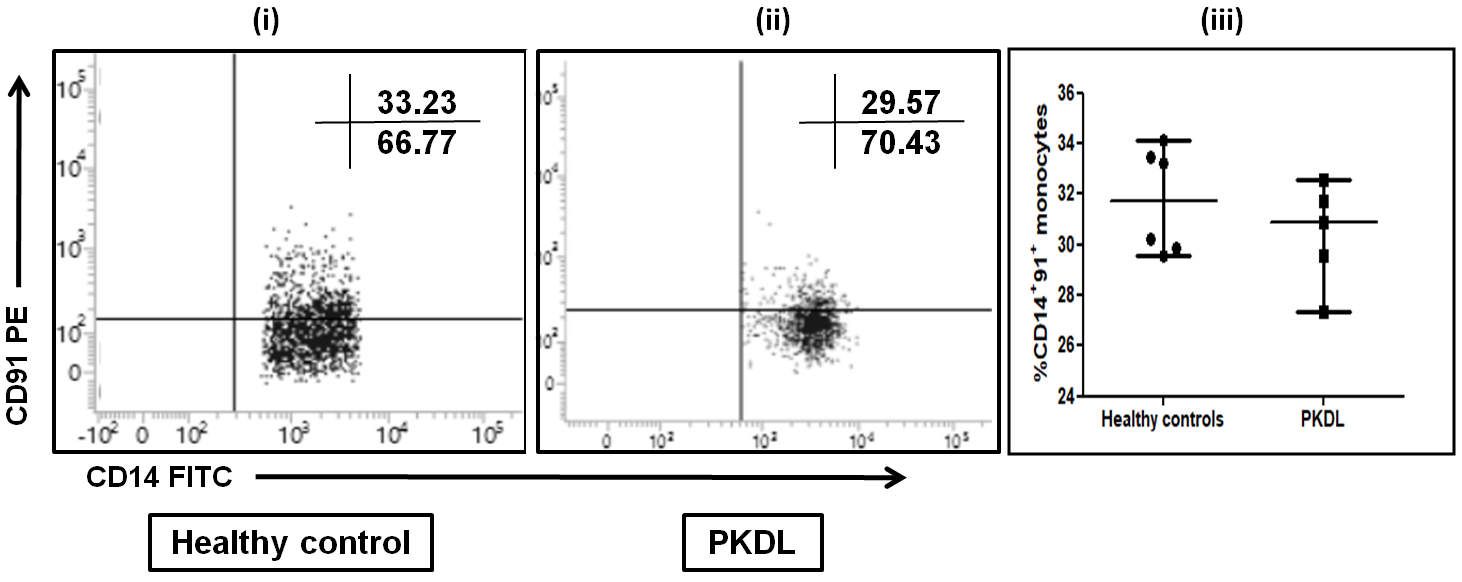

Supplement: S1 Fig — Individual gates were set by using monocyte forward and side scatter characteristics and then with fluorochrome conjugated CD14-FITC; (iii) scatter plots indicating frequency of CD91+ within CD14+ monocytes in healthy controls (n = 6, black filled circle) and patients with PKDL at presentation (n = 5, black filled square);each horizontal bar represents the median. (TIF) [file pntd.0007991.s002.tif]

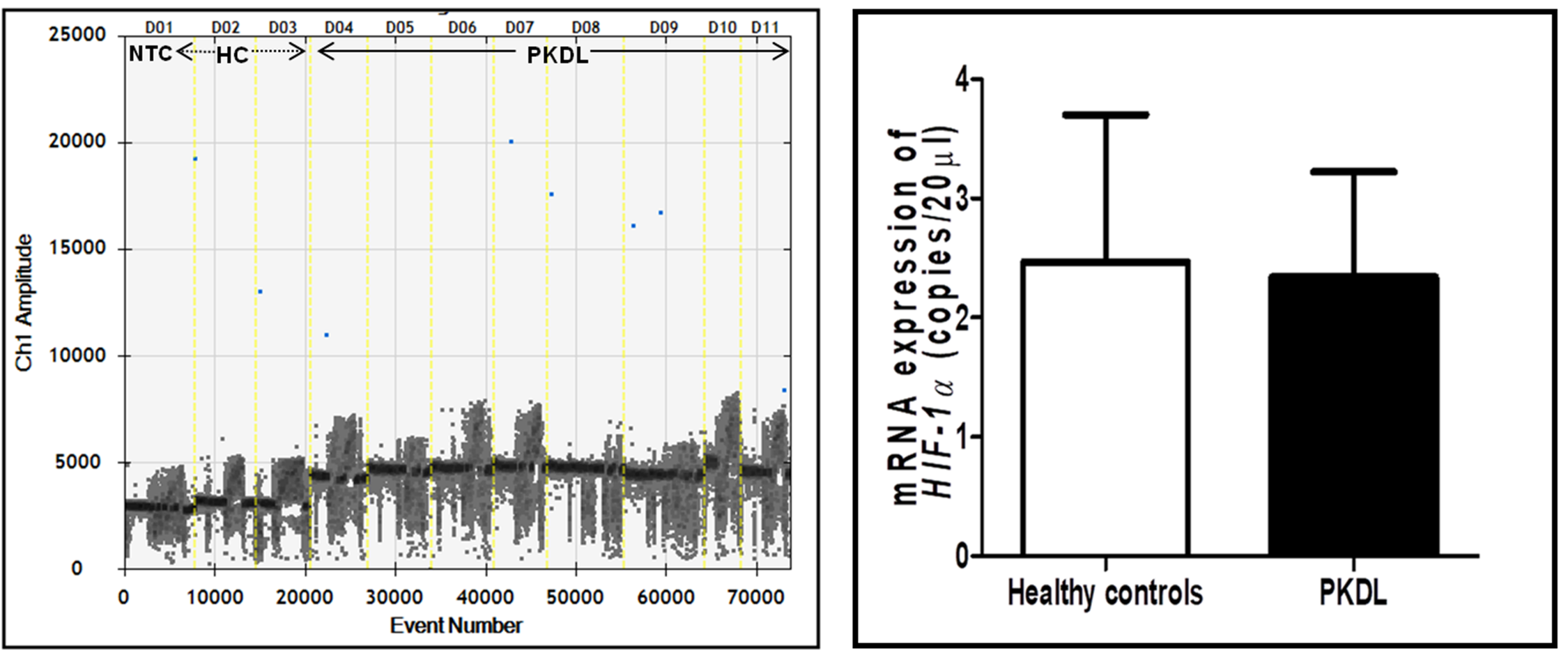

Supplement: S2 Fig — EvaGreen-bound positive droplets are shown in blue while negative droplets are shown in black, along with bar graphs for data expressed as mean ± SEM of the copy number/20 μl DNA Bar graphs (open) denote healthy controls (n = 2) while filled bars represent patients with PKDL (n = 8). (TIF) [file pntd.0007991.s003.tif]

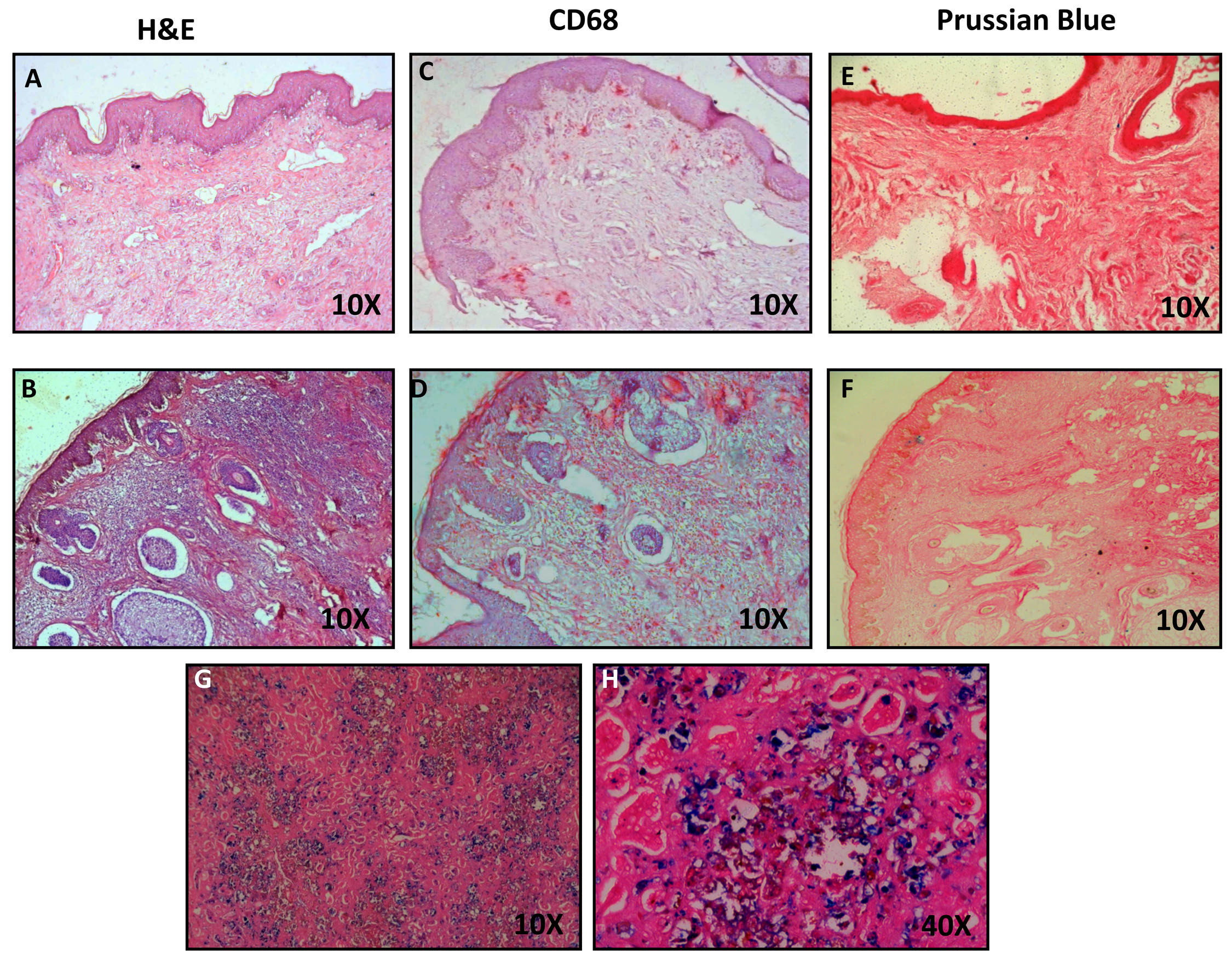

Supplement: S3 Fig — A&B: Representative H&E profiles from dermal biopsies of a healthy control and patient with PKDL (magnification 10X).C&D: Representative immunohistochemical profiles of CD68+ macrophages from dermal biopsies from a healthy control and patient with PKDL (magnification 10X). E&F: Representative Prussian blue stained profiles from dermal biopsies of a healthy control and patient with PKDL (magnification 10X) showing absence of free ferric ion. G&H: Positive control (Hemochromatosis liver section; magnification 10X and 40X). (TIF) [file pntd.0007991.s004.tif]
