# Supplementary material for: Iron trafficking in patients with Indian Post kala-azar dermal leishmaniasis
Source: PLoS Negl Trop Dis. 2020 Feb 5;14(2):e0007991. doi: 10.1371/journal.pntd.0007991 (PMC7001907; doi:10.1371/journal.pntd.0007991)
Supplement: Attachment — Submitted filename: review.docx [file pntd.0007991.s005.docx]

The manuscript is on iron trafficking in macrophages in PKDL cases. The authors report enhanced iron influx through CD163, TfR, Lcn-2 and DMT-1 probably causing increase in labile iron pool in the macrophages/monocytes. They also report enhanced expression of iron exporters like-NRAMP-1 and Fpn-1 thus defying the classical characteristics (Ferr low/ fero high) phenotype of M2 macrophages. But there are some major points that required to be addressed which are as follows:

1. The group showed M2 polarization of macrophages in PKDL cases earlier. The observation of the authors ferr high/fero high needs to be addressed and explained since it deviates M2 characteristics of macrophages. The regulation of ferritin and feroprotin needs to be addressed in PKDL.
2. Since these macrophages defies a classical phenomenon of M2 macrophages, the hallmark cytokines for M2 polarization should be reported.
3. The serum hemoglobin and iron level should be measured.
4. The hepcidin is known to play a role in ferroportin expression. Thus, the level of hepcidin should be studied to support the observation of high ferroportin expression.
5. HIF are major regulator of a number of genes involved in iron homeostasis. The precise involvement of HIF regulation in PKDL requires investigation.
6. There are no comments on CD91 expression that transports hemopexin playing important role in iron trafficking.
7. The amount of ROS generation should be shown in support of increased HO-1 expression.
